# Chronic urine ascites secondary to proximal migrating of double-J ureteral stent into peritoneal cavity: A case report

**DOI:** 10.1016/j.eucr.2022.102233

**Published:** 2022-09-16

**Authors:** Surawach Piyawannarat, Yada Phengsalae, Chinnakhet Ketsuwan

**Affiliations:** Division of Urology, Department of Surgery, Faculty of Medicine Ramathibodi Hospital, Mahidol University, Bangkok, Thailand

**Keywords:** Double-J stent, Urine ascites

## Abstract

Implanting of a prophylactic double J stent during ureteroneocystostomy has been adopted as routine procedure for preventing anastomotic complications. In extremely rare events, the coiled distal end of the stent migrates upward through ureterovesical anastomosis into the peritoneal cavity. We report below a case that presented with chronic urinary ascites secondary to ureteral stent displacement which was successfully treated by endoscopic intervention.

## 1 Introduction

Iatrogenic ureteral injury is potentially a catastrophic adverse event requiring intra-abdominal surgery, especially in gynecologic, colorectal, and vascular procedures, and it increases the risk of hospital readmission.^1^ Most operative ureter injuries occur in the distal part. An ureteroneocystostomy with double-J (DJ) stent insertion is an ideal option for this repair. The stent mimics a foreign body causing minor consequences, including irritated voiding, suprapubic pain, and urinary tract infection, but serious complications, such as spontaneous upward distal coil migration into the peritoneal cavity, are rarely reported. This interesting clinical presentation and rationale for management are discussed below.

## 2 Case report

A 50-year-old female patient was referred to our urological department due to chronic weight loss and large ascites. Three months previously, she had undergone an open abdominal hysterectomy due to severe diffuse uterine adenomyosis and concurrent ureteral reimplantation from an accidental right distal ureter injury at another hospital. The overall surgical procedure was smooth, and the immediate postoperative course was uneventful. Nonetheless, during a follow up with the primary surgeon, she was gradually run down with unexplained weight loss, persistent low-grade fever, and abdominal discomfort, so she therefore required a second medical opinion. On examination, the patient was lethargic, dehydrated, and had abdominal distension with shifting dullness. Her hemodynamic parameters were normal except for elevated body temperature (38.2 °C). Additionally, blood tests revealed leukocytosis with a leukocyte count of 13.2 × 10^5^/m and neutrophils of 84.6%. Her serum creatinine was slightly elevated from 0.5 to 1.0 mg/dl, whereas urine analysis showed increased leukocytes and red cells.

**Fig. 1:**
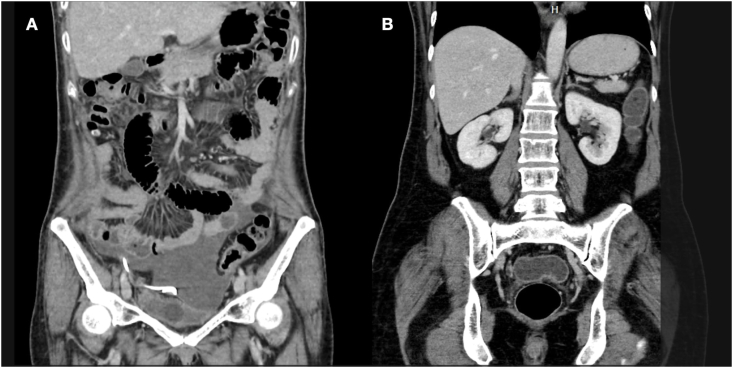
A, Pre-operative computerized tomography scan showing the distal end of the double-J stent placed outside the bladder with large ascites; B, repeated computerized tomography scan taken at 6 months showing no hydronephrosis.

An abdominal computerized tomography revealed massive ascites fluid and the distal tip of the ureteric stent within the peritoneal cavity (Fig. 1A). Abdominal paracentesis was done with a yield of 2.8 L straw-colored fluid. An ascitic fluid study revealed a high creatinine level of 5.76. These clinical data raised suspicion of intraabdominal urinary leakage. We therefore applied a nephrostomy tube and administrated intravenous antibiotics (ampicillin plus gentamicin). Two weeks later, her clinical condition had improved from sufficient nutrition. Surgical options were discussed, and it was concluded that endoscopic surgery should be performed.

**Fig. 2:**
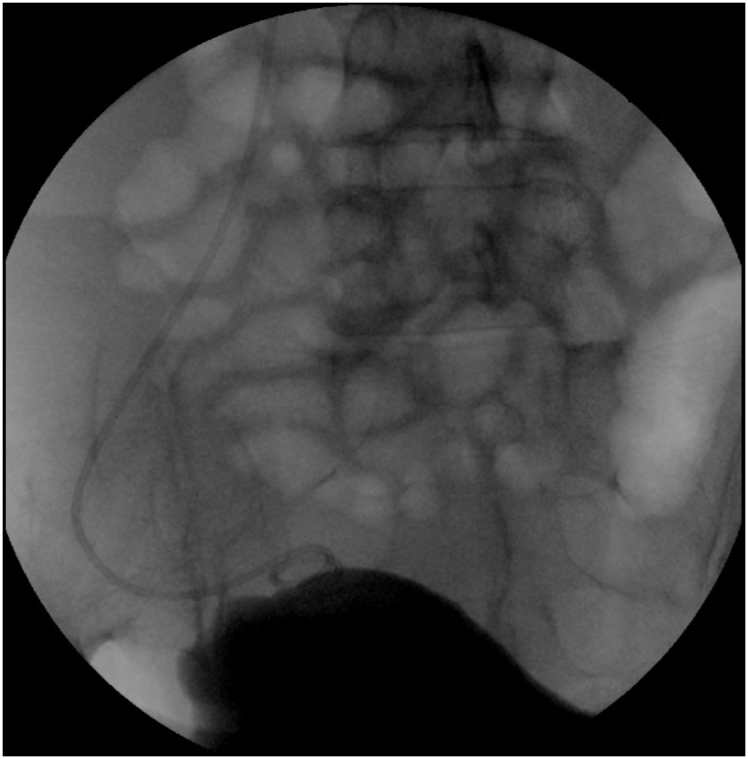
Cystography with upward migration of the stent.

**Fig. 3:**
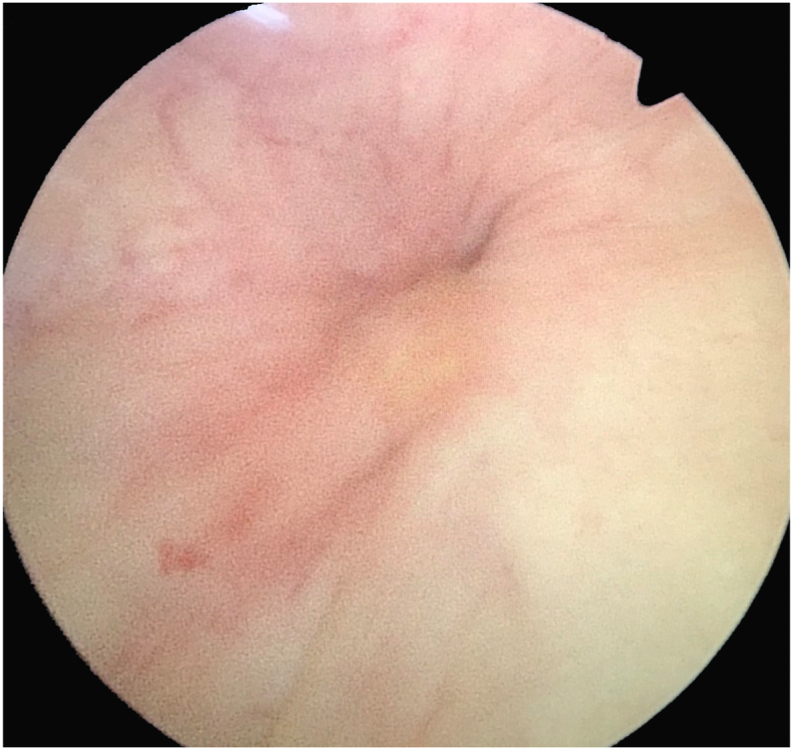
Suspicious area of migration from cystoscopy view.

The patient was placed in the dorsal lithotomy position. Retrograde cystography imaged the bladder and the location of the stent (Fig. 2). Rigid cystoscopy was applied to carefully inspect for abnormal intravesical findings and bladder mucosa was incised in suspicious areas (Fig. 3) under direct vision and with fluoroscopic guidance. Tissue cutting was performed using a 20 W holmium laser with a 550 μm core laser fiber at a power of 1 J and a rate of 10 Hz. Graper forceps were used to pull the discovered distal tip DJ stent into the intravesical space. The patient made an uneventful postoperative recovery. The stent was removed cystoscopically at 3 months after discharge. The renal scan and repeated CT scan images taken at 6 months (Fig. 1B) illustrated no hydronephrosis, and kidney function was preserved.

## 3 Discussion

Intraoperatively recognized ureteral injuries remain a challenge. Prompt identification and correction of urinary tract defects are mandatory to achieve excellent postoperative results and preventing late complications. Unrecognized injuries or delayed diagnosis can cause prolonged morbidity leading to fistula formation, impaired renal function, or even mortality. Ureteroneocystostomy is the procedure of choice to manage distal ureteral injuries that occur in close proximity to the bladder. The surgical technique comprised a non-refluxing ureteral implantation by creating a submucosal tunnel or a refluxing non-tunneled anastomosis in cases where the ureter length is inadequate for tunneling. Anastomotic leakage after repairing an iatrogenic ureter injury is one of the major concerns, yet the incidence is rare.^2^ Various fundamental principles are necessary to prevent this leakage. First, it is important to debride all necrotic tissue and the ureteral adventitial layer must be preserved to guarantee adequate arterial supply. Second, the ureter should be sufficiently mobilized to permit a tension-free anastomosis and the distal ends of it should be spatulated to increase the anastomotic surface. Finally, a water-tight, tension-free, mucosa-to-mucosa anastomosis should be generated over a DJ stent using an absorbable suture. Another concern is proximal migration of DJ stent which can be prevented by using an appropriate length that is correctly deployed with satisfactory positioning.

Urinary ascites is a very rare condition that can lead to several life-threatening complications. It may arise from nontraumatic and traumatic etiologies. Nontraumatic causes are as a result of direct invasion of closing aggressive tumors, such as cervical and rectosigmoid malignancy. Traumatic causes can be inadvertent urinary organ injury from pelvic surgery or penetrating abdominal injuries. A patient previously in good physical condition and unexplained ascites with reduced kidney function and a recent history of pelvic surgery should therefore raise the suspicion of intraperitoneal urinary leakage. Long-term intraperitoneal urine accumulation induces systemic absorption of toxic metabolites and an increased risk of developing diffuse peritonitis from bacterial organisms. A surprisingly high creatinine level in the ascitic fluid is indicative of urinary ascites, especially if the ascitic fluid creatinine-to-serum creatinine ratio is greater than 1.^3^

Currently, the management of misplaced stents consists of open surgical techniques, interventional radiological techniques, and endourologic management, depending on the location of the stent retained, the presence of intraabdominal adhesions, and surgical expertise.^4^^,^^5^ In our case, the upper part of the stent appeared to be in the proper renal pelvis position, while the lower part was closely attached to the bladder serosa. Moreover, long-term urine irritation can lead to highly inflammatory processes and formation of intra-abdominal adhesions that may severely affect difficult abdominal access and distorted anatomy for open abdominal procedures. Therefore, it was possible and feasible to safely attempt endoscopic surgery. We confirmed this achievement with excellent imaging results during the follow-up period.

## 4 Conclusion

Urinary ascites is an essential clinical entity to recognize, as it can be immediately identified and corrected. This is the first presentation of malignant urine ascites secondary to a proximal migrated ureteric catheter that was successfully treated by endoscopic intervention.

## Declaration of competing interest

None.
